# Multicenter study on long-term growth in patients with phenylketonuria

**DOI:** 10.1186/s13023-025-03946-3

**Published:** 2025-08-04

**Authors:** S. Stanescu, A. Belanger-Quintana, J. C. Rocha, M. F. Almeida, K. Ahring, K. Dokoupil, A. M. Lammardo, E. van Dam, A. Muriel, A. MacDonald, on behalf of the ENEP group, A. Belanger-Quintana, K. Dokoupil, K. Ahring, J. C. Rocha, H. GokmenOzel, M. Robert, E. van Dam, A. M. Lammardo, A. MacDonald

**Affiliations:** 1 U. Enf. Metabolicas, Paediatric Department, MetabERN, Hospital Universitario Ramón y Cajal, Madrid, Spain; 2 Nutrition & Metabolism, NOVA Medical School, Faculdade de Ciências Médicas, NMS, FCM, Universidade NOVA de Lisboa, Lisbon, Portugal; 3 CINTESIS@RISE, Nutrition and Metabolism, NOVA Medical School, Faculdade de Ciências Médicas, NMS, FCM, Universidade NOVA de Lisboa, Lisbon, Portugal; 4 CHRC, NOVA Medical School, Faculdade de Ciências Médicas, NMS, FCM, Universidade NOVA de Lisboa, Lisbon, Portugal; 5 Centro de Referência Para As Doenças Hereditárias Do Metabolismo, Unidade Local de Saúde de Santo António, Porto, Portugal; 6 Centro de Genética Médica Jacinto Magalhães, Unidade Local de Saúde de Santo António, Porto, Portugal; 7 Unidade Multidisciplinar de Investigação Em Biomedicina, Instituto de Ciências Biomédicas Abel Salazar, Universidade Do Porto, Porto, Portugal; 8 Laboratório Para a Investigação Integrativa E Translacional Em Saúde Populacional (ITR), Universidade Do Porto, Porto, Portugal; 9 Center for PKU, Kennedy Institute, Glostrup, Denmark; 10 Dp Metab Nutr, Dr Hauner Child Hosp, Munich, Germany; 11 Dp Pediatric, San Paolo Hosp, Univ Milan, Milan, Italy; 12 S. Metab Dis, Univ Med Centre Groningen, Groningen, Netherlands; 13 U. Bioestadística Clínica, Instituto Ramón y Cajal de Investigación Sanitaria, Hospital Universitario Ramón y Cajal, Madrid, Spain; 14 Dietetic Department, Birmingham Children’s Hospital, Birmingham, UK; 15 Center for PKU, Copenhagen University Hospital, Copenhagen, Denmark

**Keywords:** Phenylketonuria, Growth, Diet, Natural protein intake

## Abstract

**Introduction:**

Most of the studies on PKU have focused on the neurological development of patients. Studies regarding the physical development usually cover a short period of time, do not include dietary information, and results are contradictory. The aim of this study is to determine whether the patients with PKU have a normal growth, the incidence of obesity and the relationship of these parameters with diet and nutritional intake.

**Material and methods:**

This is a retrospective, multicenter, multinational study including patients with PKU from 8 centers from different countries. Data of growth parameters and dietary regimes were collected from birth until the age of 18 years. Anthropometric tools of the WHO (Anthro version 3.2.2 and Anthro plus) were used to calculate z-score for weight-for-age, height-for-age and body mass index (BMI).

**Results:**

Data from 182 patients with classical PKU were included. The median height z-scores for both male and female patients showed a normal growth pattern according to the WHO charts. Significant positive correlation  was observed between height z-score and the Phe (mg/day) and natural protein (g/day) intakes in all ages, especially in children younger than 11 years. The amount of Phe-free amino acid mixture did not affect the height, but lower intakes were negative correlated with the BMI. Also, we detected a positive correlation between the median Phe levels and BMI, meaning that the poorer metabolic control was correlated with higher BMI.

**Conclusions:**

An objective of PKU is that patients have satisfactory final physical development; the height prognosis seems to be associated with Phe and natural protein intake and therefore should be optimized. It is important to collect longitudinal growth data throughout childhood and adolescence in PKU that considers any change in growth in relationship to dietary patterns.

**Supplementary Information:**

The online version contains supplementary material available at 10.1186/s13023-025-03946-3.

## Introduction

PKU is a rare, autosomal recessive inborn of phenylalanine (Phe) metabolism characterized by the deficiency of phenylalanine hydroxylase (PAH) that converts the Phe into tyrosine. The PAH deficiency causes high plasma Phe level and if untreated leads to permanent neurological damage [1]. It is detected by newborn screening and the treatment for most patients is mainly dietetic, consisting in a low Phe intake in combination with Phe-free L-amino acid supplements and ingestion of special low protein foods [2, 3]. The residual enzyme activity determines the amount of Phe that a patient can tolerate to maintain the plasma levels within an acceptable range according to guidelines [3]; the tolerated Phe in the diet together with the Phe levels in the neonatal screening define the patient phenotype.

The main goal of dietary phenylalanine restriction is to achieve normal neurological development. Dietary treatment is successful at preventing neurological impairment, but the physical development of patients is given less attention. Some studies detect normal growth [4–10], while other report suboptimal outcomes [11–14]. Also, it has been proposed that in patients with PKU, low physical activity [15] and a higher energy content of the low-protein foods [16] are contributing factors of positive energy balance and obesity, especially in those with poor metabolic control [15]. It is however unclear which fraction of the diet has the strongest influence on the physical development [17].

Our study aimed to determine if a Phe restrictive diet affects the long-term growth in patients with PKU compared to the normal population and to investigate the impact of the diet on different variables of the physical development.

## Material and methods

This is a retrospective, multicenter, multinational study that includes data from patients with a classical PKU phenotype being monitored since diagnosis in the newborn period from 8 centers in different countries (Belgium, Denmark, Germany, Nederland, Portugal, Turkey, Spain, UK). Patients with milder forms of the disease or having any kind of pharmacological treatment and those that did not complete the follow-up until the 18 years old were excluded. Data of growth parameters and dietary regimes were collected annually from birth until the age of 18 years. When possible, their parents’ height was obtained in order to calculate their expected genetic height.

Weight, height and BMI were determined at diagnosis and annually thereafter. Anthropometric tools from the World Health Organization (WHO) (Anthro version 3.2.2 and Anthro Plus) were used to calculate weight-for-age, height-for-age and body mass index (BMI) z-scores. We defined overweight as a BMI z-score of > + 1 and obesity as a z-score of > + 2 for patients between 5 and 18 years old; for children younger than 5, BMI between 2 and 3 was indicated overweight and BMI > 3 obesity. The normal height was defined as a z-score between − 2 and + 2 SD [18].

Follow-up and dietary interventions were retrospectively collected from medical records and the local patient's registry. Median Phe (mg/day), natural protein (g/day), total protein (g/kg/day) and amino acids mixture (g/kg/day) intakes data were calculated annually. All the anthropometric and nutritional data were collected closest to the patients´ birthday.

Continuous variables are presented as mean and standard deviation, while categorical variables are shown using absolute and relative frequencies. To analyze the association between variables, a Generalized Estimating Equations (GEE) model with an autoregressive structure of order 1 (AR-1) was employed, as multiple measurements were taken from the same patient. All analyses were performed using Stata version 18.

## Results

Data from 182 patients with a classical PKU phenotype (100 female and 82 male) from different European countries and Turkey centers were collected and findings are presented as a group (see Supplementary Table 1).

Table 1 summarizes the mean dietary prescriptions, and the blood Phe control of the patients included. The mean total protein intake, defined as the addition of natural protein and the amino acid mixture, was higher than the WHO safe level of protein intake, considering the contribution of the special amino acids mixture [3]. Mean dried-blood spot Phe levels were generally slightly higher than the age-specific currently recommended range [3], although at the time of the recollection sample, they met local guidelines.

**Table 1:** Mean nutritional prescriptions and the blood phenylalanine control

Age (years)	1	2	3	4	5	6	7	8	9	10	11	12	13	14	15	16	17	18
Prescribed Phe intake (mg/day)																		
N	167	172	169	173	175	174	178	174	176	178	172	174	171	169	167	158	155	151
Mean	314	360	387	402	410	433	445	457	474	495	517	528	567	612	615	665	685	781
Std. Dev	123	150	157	159	165	177	178	189	186	224	224	250	215	319	252	353	324	473
Prescribed natural protein intake (g/day)																		
N	172	176	171	175	176	176	178	174	176	177	173	176	173	170	100	158	155	156
Mean	6	7	7	7	8	8	8	9	9.5	10	10	10	11	11	12	13	13	14
Std. Dev	2.6	3.1	3.2	3.2	3.3	3.6	3.5	3.8	3.7	4.4	5.0	4.0	4.4	4.9	5.2	6.9	6.5	9.7
Prescribed Phe- Free formula intake (g/kg/day)																		
N	135	139	137	137	138	140	142	139	140	140	136	140	138	132	136	130	133	129
Mean	2.04	2.07	1.20	1.82	1.77	1.63	1.55	1.49	1.45	1.38	1.28	1.20	1.15	1.08	1.03	1.01	0.99	0.96
Std. Dev	0.65	0.68	0.63	0.63	0.65	0.59	0.57	0.58	0.54	0.54	0.51	0.47	0.45	0.43	0.43	0.40	0.38	0.38
Median blood Phe levels (umol/L)																		
N	175	177	178	177	180	181	82	181	181	181	181	180	180	180	178	175	173	167
Mean	295	345	371	406	410	408	449	436	454	483	511	545	583	610	667	683	672	710
Std. Dev	151.4	170.0	20.3	245.5	228.0	228.2	398.8	264.3	237.7	252.6	266.6	261.4	281.2	285.0	298.9	278.6	299.5	322.8

**Fig. 1:**
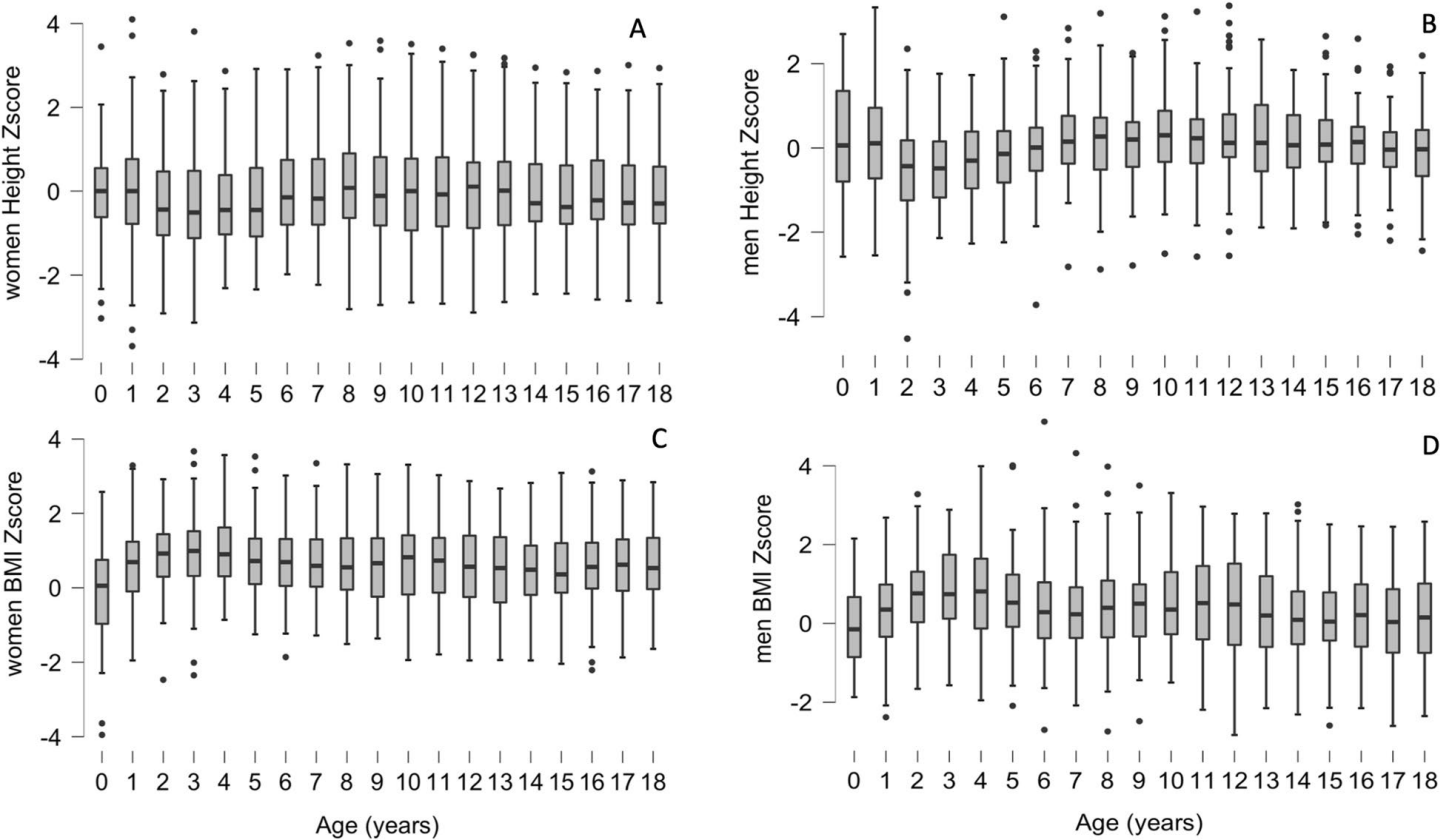
represents the height and BMI evolution over the 18 years of life of the patients included

The median height z-scores for both male and female patients showed a normal growth pattern according to the WHO charts (see Fig. 1). Genetic height data was obtained for 96 patients and 76/90 (84,4%) of the patients reached their final genetic height. BMI z-score was in the upper normal range, especially in females, since early infancy. At 18 years of age, BMI was available for 130 patients (n = 74 female; n = 56 male). Overweight was reported in 19/74 female patients (25.6%) and in 12/56 male patients (21.4%) at the age of 18 years old. Obesity was reported in 8/74 female patients (10.8%) and 2/56 male patients (3.5%).

A significant positive correlation was found between height z-score and the Phe (mg/day) and natural protein intake (g/day) in all ages. This was especially significant in smaller children (< 11 years old) and women. The amount of Phe-free mixture recommended did not affect the height in our study. However, we detected a significant negative correlation on the BMI at all ages, suggesting that the patients with a lower recommended intake of special formula had a higher BMI. Similarly, we found that there is a positive correlation between the median blood Phe levels and BMI, meaning that the poorer metabolic control was correlated with higher BMI (see Table 2).

**Table 2:** Contains the correlations between anthropometric measures and dietary parameters. Phe, phenylalanine; NP, natural protein; AA Mix, amino acid mixture

*All patients*					
	BMI z-score	*p* value		Height z-score	*p* value
Phe intake (mg/day)	0.00002(− 0.00014; 0.00018)	0.811	Phe intake	0.00022(0.00008; 0.00036)	*0.002*
NP intake (g/day)	− 0.00045(− 0.00833; 0.00743)	0.911	NP intake	0.01150(0.00461; 0.01839)	*0.001*
AA Mix (g/kg/day)	− 0.17380(− 0.25212; − 0.09548)	*0.000*	AA Mix	− 0.04409(− 0.11239; 0.02422)	0.206
Median blood Phe level (umol/L)	0.00035(0.00022; 0.00047)	*0.000*	Median blood Phe level	− 0.00009(− 0.00020; 0.00003)	0.130
< = *11 years*					
Phe intake	− 0.00006(− 0.00022; 0.00011)	0.502	Phe intake	0.00026(0.00010; 0.00043)	*0.001*
NP intake	− 0.00614(− 0.01407; 0.00179)	0.129	NP intake	0.01345(0.00581; 0.02109)	*0.001*
AA Mix	− 0.37163(− 0.53143; − 0.21183)	*0.000*	AA Mix	− 0.02446(− 0.18005; 0.13113)	0.758
Median blood Phe level	0.00032(0.00013; 0.00051)	*0.001*	Median blood Phe level	− 0.00004(− 0.00021; 0.00014)	0.683
> *11 years*					
Phe intake	0.00054(0.00015; 0.00093)	0.007	Phe intake	0.00005(− 0.00029; 0.00038)	0.782
NP intake	0.02673(0.00752; 0.04594)	0.006	NP intake	0.00161(− 0.01493; 0.01815)	0.849
AA Mix	− 0.21665(− 0.31390; − 0.11939)	*0.000*	AA Mix	− 0.03011(− 0.11500; 0.05478)	0.487
Median blood Phe level	0.00027(0.00009; 0.00045)	*0.004*	Median blood Phe level	0.00003(− 0.00013; 0.00019)	0.701
*Females*					
Phe intake	0.00005(− 0.00018; 0.00029)	0.665	Phe intake	0.00034(0.00014; 0.00054)	*0.001*
NP intake	0.00180(− 0.00961; 0.01321)	0.757	NP intake	0.01663(0.00701; 0.02626)	*0.001*
AA Mix	− 0.23611(− 0.33928; − 0.13294)	*0.000*	AA Mix	− 0.07618(− 0.16654; 0.01418)	0.098
Median Phe level	0.00028(0.00013; 0.00043)	*0.000*	Median blood Phe level	− 0.00008(− 0.00021; 0.00005)	0.223
*Males*					
Phe intake	0.00002(− 0.00021; 0.00024)	0.881	Phe intake	0.00008(− 0.00013; 0.00029)	0.465
NP intake	− 0.00124(− 0.01232; 0.00984)	0.826	NP intake	0.00518(− 0.00497; 0.01533)	0.317
AA Mix	− 0.08652(− 0.20752; 0.03448)	0.161	AA Mix	− 0.01722(− 0.12036; 0.08592)	0.744
Median blood Phe levels	0.00050(0.00027; 0.00074)	*0.000*	Median blood Phe level	− 0.00011(− 0.00032; 0.00011)	0.329

*Phe* phenylalanine; *NP* natural protein; *AA Mix* amino acid mixture

## Discussion

This study highlights the impact of dietary management on the physical development of the children and adolescents with PKU. The growth in PKU patients is expected to be normal according to the WHO, but more importantly, we demonstrate how the different components of the nutritional treatment may influence various aspects of the somatic development of patients: the natural protein and Phe intakes strongly correlate with height, while the Phe-free amino acids and the poor metabolic control appear to have a stronger impact  on the BMI. It is one of the largest studies conducted so far studying the interplay between dietary restrictions and physical development in patients with PKU. It is also one of the few studies that is multicentric and multinational and therefore includes patients that have received different dietetic recommendations.

Treatment in PKU is mainly centered on the neurocognitive outcomes. Historically, the goal in infants and children was to lower plasma Phe levels to ‘physiological concentrations’ but this led to severe restriction of natural protein [19, 20]. Together with the inadequate composition of the Phe-free amino acid supplements, this very restrictive diet led to reported malnutrition, anorexia, and impaired growth [21, 22]. Since the early days of treatment, there has been important progress made in understanding the Phe requirements and improvement in the composition of the free Phe amino acid supplements in PKU [23].

Optimal dietary management is essential in producing normal growth during childhood. Chronic disease or malnutrition have a deep impact on the physical development. In this regard, patients with inborn errors of metabolism, like organic acidemia or urea cycle disease together with other aminoacidopathies such as PKU, treated with protein restriction and synthetic formulas, have a significant risk for growth retardation [24, 25]. In other words, the mismatch between patient’s nutritional needs and nutrient prescribed/delivered at critical moments throughout pediatric age, may have contributed to these suboptimal outcomes.

The reasons for impaired growth in PKU are unclear and there are important uncertainties regarding the impact of the different dietary components on physical development [14, 26]. Fear of neurological symptoms and the desire for optimal blood Phe control may lead physicians and families to follow a very strict diet during the first years of life. It has been proposed that poor growth is associated with a severe Phe restriction [22] and that a higher natural protein intake is associated with better outcome [12]. It is important to note that patients with mild forms of hyperphenylalaninemia who receive a less restrictive diet do not have growth impairment [4, 11]; this may indirectly suggest that natural protein intake is a major player in the physical development of children with PKU. Our results indicate a clear positive impact of the Phe/natural protein intake on height z-score. This correlation appears stronger in younger patients (< 11 years old), suggesting the important effect of the natural protein in the somatic development especially in the first years of life and the importance of challenging with natural protein intake until maximum Phe tolerance is achieved in this group of age. In this regard, it has been proposed that Phe tolerance might increase in PKU patients during certain developmental periods such as pubertal growth [27].

The Phe-free amino acids supplements are another important pilar in successful dietary management and are essential to prevent protein or nutrient deficiency and optimize metabolic control. Total protein insufficiency, rather than only natural, could also be an important reason for an impaired growth [9, 28–30], and some authors suggest more emphasis on optimizing the intake of Phe-free amino acids supplements to ensure an adequate nutritional status and to achieve normal physical development. The 2017 European guidelines recommend providing an additional 40% of L-amino acids to compensate for losses due to digestibility and to help optimize blood Phe control [3]. Still there is no uniformity in prescribing the amount of special formulas [31] and the studies that investigate the adequate intake of protein intake in PKU are limited [32]. The high amino acid mixture intake helps to promote better PKU control due its functional effect [9, 29, 33], but there are doubts regarding the bioavailability of the special formulas [34, 35]. Although there has been a huge advance in the last decades, the nutrient composition of Phe-free protein substitute raises concerns. It is documented that children who received casein hydrolysate during childhood had a significantly lower height [11]; therefor it was hypothesized that the composition of the Phe-free protein substitute has an important impact on the physical development [7]. Still, the studies that correlate the Phe-free amino acid supplements with height are few and inconsistent [29]. Our data do not suggest a significant impact of the Phe-free amino acids mixture on height. This may be related to the fact the administration of the amino acid mixture induces a rapid increase in plasma amino acid levels that exceeds the capacity of anabolic processes to incorporate them into nascent proteins [36].

Another interesting result in our study is the effect of the metabolic control on the BMI. In this regard, we observed a positive correlation between the BMI and the median blood Phe levels especially in the female cohort. There was also negative correlation of the Phe-free amino acid supplement intake on the BMI. Apart from the functional effect on the metabolic control as discussed before, the medical food intake might also have an important input in acquiring satiety and therefore reducing the need for special low protein food rich in carbohydrates. Our results are similar to previous studies, proposing that low physical activity and a higher energy content of the low-protein foods [16] are contributing factors of positive energy balance and obesity in PKU, especially in those with poor metabolic control [15, 37–39].

We acknowledge that our study has some important limitations. Being a retrospective study, not all data could be recovered or standardised. Also, as we wanted to follow patients throughout their entire childhood development, dietary recommendations and the special formula compositions have changed over time and findings, especially regarding small children, might be similar but not the same to present ones. We also used the data recorded, which mainly included dietary recommendations but not real-life intake evaluations. In young children, parents usually ensure that treatment is followed, and recommendations are similar to dietary inventories. In adolescents they might differ greatly, and our study cannot detect those cases; these individuals face a range of PKU-specific challenges, many of which can significantly compromise metabolic control [40]. However, the large group of cases and the inclusion of patients from different centers with different recommendations we believe compensates for these limitations.

A further limitation of this study is the lack of a control group, as well as the use of WHO growth charts, which are based on the general population. Somatic development is significantly influenced by genetic factors, which vary across populations. Additionally, the worldwide distribution of pathogenic PKU variants suggests that racial and ethnic differences may hold clinical relevance [41]. Nevertheless, a detailed exploration of genetic variability falls beyond the scope of the present analysis.

In conclusion, our study emphasizes that children and adolescents with PKU can and should have a normal growth. Notably, different components of the diet have a distinct impact on the somatic development. The natural protein intake has a deep impact on height, especially in younger children, while Phe-free amino acid supplements intake has significant correlation with the  BMI. These factors should be considered during the diet planification in PKU patients, and the natural protein intake should be optimized according to the individual Phe tolerance. Additionally, a higher Phe-free amino acid supplements together with a better metabolic control are associated with lower BMI.

## Supplementary Information

Additional file1 (XLSX 42 KB)

## Data Availability

The datasets used and/or analysed during the current study are available from the corresponding author on reasonable request.
